# Enteric Fever
*A Lecture delivered at the London School of Clinical Medicine.


**Published:** 1912-10-19

**Authors:** Guthrie Rankin

**Affiliations:** Physician to the "Dreadnought" and the Royal Waterloo Hospitals.


### ENTERIC FEVER.*

By GUTHRIE RANKIN, M.D., F.R.C.P., Physician to the "Dreadnought" and the Royal Waterloo Hospitals.

In the wards of the "Dreadnought" Hospital we have frequent opportunities for directing our attention to the study and treatment of enteric fever. The summer and autumn terms are those in which most cases come to us and this is in accordance with what we know of the seasonal prevalence of the disease. Those of you who participated in our work during last session will remember that we had a considerable number of cases to deal with; enough to enable us to demonstrate most of the leading features of the malady and to bring home the difficulties encountered in its recognition, especially during the early stages of the illness; the possibilities of relapse; and the variety of complications by which it may be attended.

### Early Investigations.

Enteric or typhoid fever appears to have been known in the days of Hippocrates, but it was not until 1820 that Bretonneau first described the intestinal lesion and related it to the disease; subsequently the researches of Trousseau, Jenner, and others enabled it to be clearly differentiated from typhus fever, of which, until then, it was regarded as a variety.

Early investigations led to the conclusion that it was a filth disease capable of transmission by water, milk, or food, but the theory held at one time by Murchison and other observers, that its origin was probably autogenetic, was disproved by the discovery by Eberth in 1880 of a bacillus peculiar to the disease and now universally recognised as the morbific agent upon which it depends. Recent researches have shown that in cases of enteric fever Eberth's bacillus is widely disseminated throughout the tissues of the body, so that, though the evacuations are still the greatest source of infective danger, there is more risk in personal contact than was at one time believed.

Over 200 instances of the spread of enteric fever by means of water are on record as having occurred in Great Britain during the last thirty or forty years.

Whenever the water supply of a district becomes tainted with sewage, all that is required to ensure an outbreak of enteric fever is the presence of the specific germ, which, under favourable conditions, retains its vitality for long periods of time outside the human body. In the Maidstone epidemic, for instance, which broke out in September 1897, when about two thousand persons were attacked, it was proved that the outbreak had been originated by contamination of the Farleigh Spring—one of three sources of the town water supply—probably by hop-gatherers who, at the time, were camped in its vicinity. Buhl has pointed out that in Munich the level of the soil water bears a fairly constant ratio to the number of cases of enteric fever recorded; the lower the level of the subsoil water, the greater the number of cases, and vice versa. Hot and dry seasons are admittedly favourable to the prevalence of the disease, probably because there is more dust and therefore greater facilities for aerial dissemination. There are also more flies and it is easy to understand how they may act as carriers from fæces or other sources of pollution to meat, milk, and ordinary articles of diet. In the Spanish-American War of 1898 Vaughan reported that flies undoubtedly acted as carriers of infection both by way of mechanical transportation and in their digestive canal; while in the Transvaal War Tooth found some corroboration of Vaughan's experience and believed that fæcal infection was promoted by flies and also by sand storms.

Milk is a frequent source of danger, and the bacillus has been traced to such other articles of ordinary dietary as butter, lemonade, salads, oysters, cockles, etc.

Nurses, washerwomen, and others frequently contract the disease from contact with enteric-fever patients or with their soiled bed-linen or clothes. The usual mode of entrance of the virus into the human subject is by way of the digestive tract, but it is also possible for it to be absorbed through the respiratory mucous membrane by aerial conduction. Air-borne infection may also pollute drinking water or some article of dietary, and in this secondary way gain access to the body through the alimentary canal.

The relation of sewer-gas to enteric infection is probably, in the majority of instances, a secondary risk only. The bacillus is seldom present in sewer-gas, but the effect of the gas itself on those who inhale it is to lower their resisting power and so to promote all forms of germ invasion. In this connection it has been proved by Alessi that guinea-pigs after exposure to the emanations from sewer are rendered more susceptible to inoculation with a feebly virulent strain of Eberth's bacillus than control animals not so exposed.

### General Considerations.

The disease has no special relationship to poverty or overcrowding, except in so far as these conditions are most frequently associated with sanitary defects and depressed vitality. It attacks all classes of the community alike, and is met with in every quarter of the globe, though specially prevalent in temperate climates. It has been pointed out by Sandwith that in Egypt the disease is seldom absent from the European hospitals, whereas it is of rare occurrence in the wards of the Egyptian Government hospitals, which are tenanted almost exclusively by the natives of the country. Cantlie records a similar experience in China, where, he says, the disease is common among Europeans but rare among the natives.

Improvement in sanitary arrangements has, in recent years, diminished the prevalence of enteric

* A Lecture delivered at the London School of Clinical Medicine.

fever in this country. For a similar reason, perhaps, the type of the disease has also undergone considerable change, so that, taken all round, it is a much milder disorder to-day than it was twenty-five or thirty years ago.

No age is exempt from it, but the period of greatest liability would seem to be between fifteen and thirty. It is most prevalent in late summer and early autumn. Some persons, and even some families, seem more susceptible than others, and those whose occupation brings them in contact with the poison—physicians, nurses, laundresses, etc.—run special risks.

Eberth's bacillus, to whose presence and activity the disease is due, is a rod-shaped micro-organism of 2 to 4 millimetres in length, mobile, and furnished with from eight to twelve fine flagella. It grows readily on all the usual media, both at blood-heat and at the ordinary temperature of the air. It is found in the fæces, the urine, the lymphoid tissues of the intestines, the mesenteric glands, the spleen, the rose spots, and the blood. In cases where there are pneumonic complications, it is also to be found in the sputum. It is frequently present in the urine as early as the third day of the disease, but can rarely be demonstrated in the stools before the eighth or ninth day.

The incubation period of enteric fever varies within considerable limits, but the average duration may be taken as approximately twelve days. It is not unlikely that incubation may be influenced by the degree of concentration of the poison, the virulence of the germ, and the avenue by which it gains access to the economy.

### Symptoms.

The disease usually comes on insidiously, and, during the incubation period, little more may be complained of than lassitude, pains in the limbs, and nausea, together with headache and insomnia, which are early symptoms of special importance. Its initiation is sometimes announced by rigors. In the first week the malaise of the prodromal period becomes more pronounced, epistaxis is of frequent occurrence, and the patient becomes feverish and restless at night. The temperature steadily increases, the evening record being about 1° higher each day. The pulse is accelerated, of full volume, but low in tension and often dicrotic. The tongue is small and pointed, red at the tip and edges, with its papillæ unduly prominent. There is already some distension of the abdomen, with tenderness which is especially marked in the right iliac fossa, where there is gurgling on deep pressure. The headache continues, giddiness is often complained of on sitting up, and some degree of mental wandering at night may set in. The bowels are either slightly relaxed or obstinately confined. As the end of the week approaches the spleen is found to be increased in size, and the first crop of rose-coloured spots appear, usually over the lower chest and abdomen. These spots are well defined and raised above the surface, round or oval in shape, from 1 to 3 millimetres in diameter, and of a pinkish or rose colour, which disappears on pressure, but never becomes petechial. They may be few in number and easily overlooked, but, in some cases, they are so numerous as to attract immediate attention. The eruption occurs in successive crops of spots, each crop lasting about four days, and the first usually declaring itself between the sixth and twelfth day of the disease. The urine presents the usual febrile changes, but does not necessarily contain albumen. Bronchial catarrh is not an uncommon incident during the first week.

In the second week the symptoms of the first week are aggravated, and the temperature continues at a high level with only slight morning remissions. The headache, which rarely continues beyond the tenth day, is replaced by mental dulness and stupor, and there is a frequent accompaniment of deafness, with buzzing in one or both ears. The tongue becomes dry, glazed, and cracked, and sores accumulate round the teeth. The typanitis and iliac fossa tenderness increase, and there may be more or less profuse diarrhœa, though constipation continues the feature of many cases. The stools, when loose, assume the typical pea-soup colour with which we are familiar.

During the third week the temperature curve begins to show a daily variation of about 2°. Instead of remaining, as during the second week, at a fairly uniform level, it falls lower and lower each morning, while the evening record, though also declining, comes down more gradually; the morning temperature may thus, in four or five days, reach 98° or 99°, while in the evening it still touches 101° or 102°. The pulse usually ranges from 100 to 120. There is marked weakness; more or less pronounced meteorism; an increased number of stools if there be diarrhœa; and a condition of general prostration—manifested by drowsiness, low muttering delirium, muscular tremors, subsultus tendinum, carphologia, and irresponsiveness to the attentions of those in attendance—which is known as the "typhoid state." Bed-sores are apt to form over the sacrum and other prominent parts.

### Complications and Immunity.

It is during this week that pulmonary complications, cardiac failure, hæmorrhage from the bowel, and perforation are most to be feared. In the case of hæmorrhage, the blood which is evacuated will be bright if its source is an artery which has been perforated during the separation of a slough, but dark if it proceeds from a general oozing in the congested areas of the bowel. A moderate bleeding may occur without seriously disturbing the ordinary course of events, but if the amount is copious and of sudden onset, the patient becomes cold, blanched, collapsed, pulseless, and his temperature falls 2° or 3°. The most alarming feature about hæmorrhage is the possibility of its being the forerunner of perforation.

In favourable cases the fourth week is the week of convalescence. The diarrhœa, if previously present, subsides; the tongue moistens and cleans; the temperature gradually sinks to normal; and the appetite is restored or may become exaggerated.

In severe cases, the urgent symptoms of the third week continue in an aggravated degree, and convalescence may be delayed till the fifth or even the sixth week.

One attack does not confer any lasting immunity from risk of recurrence in anything like the same degree as is the rule in other infective fevers. Relapses are always to be dreaded; they occur in from 6 to 10 per cent. of all cases, and one or more may happen. They must be regarded as autogenous reinfections, and usually set in within two or three weeks after complete defervescence has been established.

This sketch describes in broad outline the course of a typical case, but there are certain deviations from the usual so important that they must be referred to.

### Deviations from the Normal.

Instead of being gradual, the invasion may be sudden and acute, with (*a*) *nervous manifestations*—delirium, meningitis, convulsions, mania; (*b*) *pulmonary symptoms*—bronchitis, pneumonia, pleurisy; (*c*) *gastro-intestinal symptoms*—uncontrollable vomiting, intense diarrhœa, intestinal hæmorrhage, perforation; (*d*) *renal symptoms*—nephritis, suppression of urine.

A very obscure and dangerous variety of invasion is that known as the *ambulatory form* of the disease, when the patient fights against the minor early symptoms of his complaint and remains at work until some acute development, or debility, compels him to take to his bed. Many ambulatory cases are responsible for the apparently acute modes of onset which have been enumerated.

When the temperature follows the usual course it climbs by a step-like arrangement during the first week, the evening record being from 1° to 1.5° higher than that of the morning. Having reached its height, which may be 104° or 105° in the evening, the fever persists during the second week with but slight morning remissions. During the third week it becomes distinctly remittent, with an alternation between morning and evening of 3° or 4°. In irregular cases the initial temperature may be high—103° or 104°—or the usual curve may be reversed and show a high morning and lower evening record.

Hyperpyrexia is uncommon, but may happen before death. Dietary error, mental upset, or physical exertion may cause a temporary rise of temperature to 102° or 103° some days after normal defervescence has taken place. The behaviour of the temperature during a relapse is a repetition of the original, but it is usually lower, and rarely persists for more than two weeks. Gerhardt has described cases in which there has been no temperature disturbance.

Fresh crops of the typical rose-rash are seldom met with after the beginning of the third week. In about one-fourth of all cases no rash is discoverable; this is most frequently the case in children. Accidental rashes—erythema, urticaria, herpes, etc.—sometimes occur in the early stages of the illness. They are probably toxæmic. Miliaria and sudamina are common in cases where there is copious perspiration. Œdema may occur, and is due either to anæmia, nephritis, or thrombosis. A peculiar odour is exhaled from the surface of the body in many patients.

Examination of the blood usually reveals a leucopenia; the large mononuclear cells are increased, while the polymorphonuclears are reduced in numbers. The red corpuscles and hæmoglobin are both below normal. The absence of leucocytosis is often a point of diagnostic importance. When the fever has spent itself there is frequently considerable anæmia to overcome. The behaviour of the pulse is important: during the first week it is full in volume, from 80 to 100, soft, and often dicrotic; it is slower, in proportion to the temperature and gravity of the illness, than in the other acute fevers; and it is conspicuously subject to alterations of volume and rhythm from the slightest causes—change of position, emotional disturbance, worry, etc.

Laryngitis, bronchitis, pleurisy, and pneumonia may all occur as incidents in the course of an attack of enteric fever. It is important to remember that pneumonia, often with obscure physical signs, may be the initial manifestation of the disease.

### An Early Symptom in Obscure Cases.

When diarrhœa occurs, it is probably more dependent upon an intestinal catarrh than upon extensive ulceration. It is generally most troublesome during and after the second week. The evacuations are thin, offensive, of pea-soup consistence and appearance; and may contain, in the third week, fragments of slough. There is always some flatulent distension of the abdomen, but extreme meteorism is to be regarded as of evil omen. Hæmorrhage from the bowel is most frequent after the second week; when it occurs earlier it is probably due to a congested state of the mucous membranes, and not to ulceration.

The spleen is enlarged, and in the majority of cases can be felt below the costal arch, sometimes as early as the beginning of the second week. The liver also may be enlarged, with an accompanying slight jaundice, but this is rare.

Severe and persisting headache is an early symptom of considerable value in obscure cases. True meningitis may occur, but is not frequent. Kernig's sign is an especially valuable guide when other suggestive symptoms declare themselves. In most patients a mild form of delirium develops during the second week. It is accompanied by a listless and apathetic attitude and by mental obtuseness. In severe cases coma-vigil may ensue. Convulsions are sometimes met with in children.

Albumen is present in about 70 per cent. of severe cases. Eberth's bacillus may be found in the urine, sometimes in such large numbers as to give the secretion a characteristic flocculent turbidity. When there is bacilluria, cystitis is apt to ensue if the urine is long retained in the bladder or if the bladder-walls get damaged.

(*To be continued.*)

